# Self-Rated Health Trajectories in the African American Health Cohort

**DOI:** 10.1371/journal.pone.0053278

**Published:** 2012-12-31

**Authors:** Padmaja Ayyagari, Fred Ullrich, Theodore K. Malmstrom, Elena M. Andresen, Mario Schootman, J. Philip Miller, Douglas K. Miller, Fredric D. Wolinsky

**Affiliations:** 1 Department of Health Management and Policy, the University of Iowa, Iowa City, Iowa United States of America; 2 Department of Neurology and Psychiatry, Saint Louis University, St. Louis, Missouri, United States of America; 3 Institute on Development and Disability, Oregon Health and Science University, Portland, Oregon, United States of America; 4 Department of Internal Medicine, Washington University in St. Louis, St. Louis, Missouri, United States of America; 5 Department of Biostatistics, Washington University in St. Louis, St. Louis, Missouri, United States of America; 6 Department of Internal Medicine, Indiana University, Bloomington, Indiana, United States of America; 7 Regenstrief Institute, Inc., Indianapolis, Indiana, United States of America; 8 Department of Internal Medicine, the University of Iowa, Iowa City, Iowa, United States of America; 9 Department of Adult Nursing, the University of Iowa, Iowa City, Iowa, United States of America; Cardiff University, United Kingdom

## Abstract

**Background:**

Self-rated health taps health holistically and dynamically blends prior health histories with current illness burdens and expectations for future health. While consistently found as an independent predictor of functional decline, sentinel health events, physician visits, hospital episodes, and mortality, much less is known about intra-individual changes in self-rated health across the life course, especially for African Americans.

**Materials/Methods:**

Data on 998 African American men and women aged 50–64 years old were taken from a probability-based community sample that was first assessed in 2000–2001 and re-assessed 1, 2, 3, 4, 7, and 9 years later. Using an innovative approach for including decedents in the analysis, semi-parametric group-based mixture models were used to identify person-centered group trajectories of self-rated health over time. Multivariable multinomial logistic regression analysis was then used to differentiate the characteristics of AAH participants classified into the different group trajectories.

**Results:**

Four self-rated health group trajectories were identified: persistently good health, good but declining health, persistently fair health, and fair but declining health. The main characteristics that differentiated the self-rated health trajectory groups from each other were age, education, smoking, morbidity (angina, congestive heart failure, diabetes, and kidney disease), having been hospitalized in the year prior to baseline, depressive symptoms, mobility limitations, and initial self-rated health.

**Conclusions:**

This is the first study to examine self-rated health trajectories separately among African Americans. Four qualitatively distinct self-rated health group trajectories were identified that call into question the accuracy of prior reports that a single, average self-rated health trajectory for African Americans adequately captures their within-group heterogeneity.

## Introduction

For four decades, self-rated health has been an important indicator capturing information above and beyond that reflected in objective health assessments and physician examinations [1]–[3]. The reason for this is that self-rated health taps a more holistic perspective of health and dynamically blends prior health histories with current illness burdens as well as expectations for future health [4]–[7]. As a result, the traditional self-rated health question–Would you say your health is excellent, very good, good, fair, or poor?–has been included in numerous studies of health and health behavior [1], [2]. Self-rated health has been shown to be an independent predictor of subsequent health events, including functional decline [8], the onset of sentinel health events [9], physician visits [10], hospital episodes [11], and mortality [5], [12].

Much less, however, is known about intra-individual changes in self-rated health across the life course [13], [14]. Some studies have shown that self-rated health is stable over time [15], other studies have shown that it improves over time [16], and still other studies have shown non-linear declines over time [17], [18]. The divergence in these findings may in part be attributable to differences in the “point of reference” used in the self-rated health question [19], [20], which can be either global (as in the traditional question shown above), self-comparative (where the question is prefaced by “Compared to your health one [or two] year[s] ago…”), or age-comparative (where the question is prefaced by “Compared to other persons your age…”). Another plausible explanation for these differential findings involves the methods used to assess changes in self-rated health over time, which have primarily focused on (a) changes between just two or three time points and thus reflect state change models rather than self-rated health trajectories *per se*, or (b) changes in the grand mean over time (i.e., estimating an average trajectory) [21], [22].

More recently, several studies have used data in which self-rated health was assessed at multiple time points and employed modeling techniques that go beyond single, average trajectory estimation. For example, Liang et al. [21] showed that self-rated health among the Japanese worsened somewhat after age 60 overall, but plateaued at about age 85. When sub-trajectories were considered, however, Liang et al. found four distinct patterns: consistently good health, early onset decline, late onset decline, and recovery. Similarly, Lee et al. [23] found that among Taiwanese older adults there were five self-rated health sub-trajectories: persistently poor, moderate declining to poor, moderate but relatively stable, abrupt declines from good to poor, and, persistently good self-rated health. Sacker et al. [24] found similar results for the United States and the United Kingdom with two persistent self-rated health trajectories–good vs. poor–and that when change occurred, it primarily reflected decline. Similar results were found when Germany and Denmark were subsequently added to the mix [25]. Finally, Liang et al. [14] found linear declines in self-rated health across Caucasian Americans, African Americans, and Hispanic Americans, but noted that the declines were greatest for African Americans. Although the declines were comparable for Hispanic Americans and Caucasian Americans, the latter had the most advantaged health at the onset. Liang et al. [14] also identified the need for future research that: (i) focused on the heterogeneity in self-rated health changes using person-centered methods that identified qualitatively distinct sub-trajectories; (ii) explored socioeconomic and sociocultural variations within race and ethnic groups; (iii) addressed the problems of left truncation and survival that may lead to selection bias; and, (iv) brought neighborhood social context more directly into the trajectory modeling process.

This study builds on the more recent investigations of self-rated health [14], [19], [21]–[29] and addresses the need for more refined and rigorous studies that fill in the knowledge gaps about self-rated health trajectories in middle and older age adults [14]. Seven strengths of this study are worth noting. First, it focuses on just one racial group–African Americans–sampled from two socioeconomically diverse geographic areas within a single metropolitan region, designed to maximize within-race contrasts. This facilitates the disentanglement of race and socioeconomic status while exploring the heterogeneity in self-rated health trajectories among African Americans. Second, this study uses seven rounds of data collection spread over nine years from the African American Health (AAH) cohort [30] to evaluate intra-individual changes. Third, it uses the Diehr et al. [9] self-rated health coding strategy of 95 for excellent, 90 for very good, 80 for good, 30 for fair, 15 for poor, and 0 for decedents. This permits incorporating attrition due to death directly into the trajectories, minimizing selection bias and approximating a simple health utility assessment. Fourth, this study uses a semi-parametric (i.e., discrete) group-based mixture modeling strategy [31], [32]. That method permits the simultaneous estimation of person-centered, multiple common (or group) trajectories. Fifth, it uses a multi-level ecological approach that incorporates neighborhood context, including self-reports, interviewer assessments, enumerator evaluations, and the local geographic area contrasts built into the AAH design. This permits bringing neighborhood social context directly into the trajectory modeling process, as advocated by Liang et al. [14]. Sixth, this study includes multiple measures of morbidity and functional status which provide more granular adjustments of index health status. Finally, this study includes multiple measures of other individual level characteristics, including demographics, socioeconomic status, attitudes and beliefs, and healthy lifestyles.

Two hypotheses framed this study. Based specifically on the findings of Liang et al. [21], Lee et al. [23], and Sacker et al. [24], the first hypothesis (**H1**) was that several qualitatively distinct intra-individual self-rated health trajectories would be identified in the AAH cohort. Moreover, we expected to find some reflecting persistent self-rated health trajectories over time, albeit at different (e.g., higher and lower) initial health states. Furthermore, we also expected to find some self-rated health trajectories reflecting declines, with these declines potentially having either early or late onset. Because of the significant disease and functional burdens facing the AAH cohort and their associated higher mortality rate [30], however, we did not expect to observe trajectories reflecting recovery. Based on previous studies [1]–[4], [6], [7], [9], [13], [14], [18], [21]–[29], the second hypothesis (**H2**) was that these qualitatively distinct self-rated health trajectories would be associated with characteristics of the AAH participants, and the context of the distinct neighborhoods in which they lived. Consistent with our previous work with the AAH cohort [30], [33], we categorized these as demographics, socioeconomic status, neighborhood characteristics, healthy lifestyles, attitudes and beliefs, morbidity, and functional status.

## Materials and Methods

### Ethics Statement

This study was fully approved by the Institutional Review Boards (IRBs) at Saint Louis University, the University of Iowa, Indiana University, Washington University, and the Oregon Health & Science University. Written informed consent was obtained from all participants at baseline, and verbal consent was obtained at all follow-up interviews.

### Sample

The AAH cohort included 998 African American men and women born in 1936–1950 whose baseline interviews occurred in 2000–2001 [30], [33]. By design, participants lived either in a poor inner-city area of St. Louis, Missouri, or in the near northwest suburbs that had mixed but generally better socioeconomic status. AAH participants in the inner city area were also more disadvantaged in terms of their illness burden and functional health status than their African American counterparts in the nationally representative Health and Retirement Study (HRS), and AAH participants in both geographic areas were more disadvantaged in their illness burden and functional health status than the Non-Hispanic Whites in the HRS [30]. Approximately equal numbers of participants were randomly recruited from each area, but because the inner city had fewer eligible persons, the probability of selection was higher there. Thus, sampling weights were used to adjust for the unequal selection probabilities as well as for the AAH sampling design. Inclusion criteria were self-reported Black or African American race and Mini-Mental Status Exam (MMSE) [34] scores ≥16, reflecting an appropriate cognitive status threshold for participating in health care decision-making, and presumably for providing reliable and valid self-reports [35]. A 76% response rate was obtained for the baseline in-home evaluations. Follow-up interviews were conducted at one, two, three, four, seven and nine years post-baseline. All predictor variables used in this study were taken from the baseline in-home evaluations, while self-rated health was taken from the baseline and all follow-up interviews. Of the 998 original participants, 582 (58%) were re-interviewed at the final follow-up for a nine-year retention rate of 67.8% among known survivors.

### Self-Rated Health

The traditional self-rated health question was used at all rounds of data collection, and asked participants “Would you say your health is excellent, very good, good, fair, or poor?” The Diehr et al. [9] coding strategy was used, with 95 for excellent, 90 for very good, 80 for good, 30 for fair, 15 for poor, and 0 for decedents. This reflects the longstanding recognition that the 1–5 response set is ordinal, with the 95-0 recoded values reflecting the empirically-derived likelihood of being in excellent health in one to two years. This recoding mirrors that in the widely used SF-36 [36]. Using this approach also incorporates the decedents (deaths were verified in the AAH by obituary review and collateral contacts identified at baseline) in the trajectory estimation after their deaths, which minimizes survival bias, and approximates a simple health utility assessment. Covariates were identified based on prior findings in the literature or recommendations from previous studies [1], [2], [14], [19], [21]–[29], and were categorized as demographics, socioeconomic status, neighborhood characteristics, healthy lifestyles, attitudes and beliefs, morbidity, and functional status. With one exception (alcohol dependency was first measured at the 1-year follow-up), the covariates were all ascertained at baseline.

### Demographics

Age, sex, and marital status captured demographic variations. Age was measured in years. Sex was coded 1 for men and 0 for women. Marital status was coded as a set of three dummy variables for divorced or separated, widowed, or never married, with married as the reference category.

### Socioeconomic Status

Education, Medicaid status, household income, and inability to afford health care captured socioeconomic status variations. Education was coded in years. Medicaid status was coded 1 for yes and 0 for no. Based on the observed distribution, income was coded 1 for having $20,000 or less annual household income and 0 for having more. Inability to afford health care was coded 1 for self-reports of not being able to see a doctor when needed due to inadequate finances and 0 for otherwise.

### Neighborhood

Geographic area, self-reported neighborhood conditions, interviewer-assessed housing conditions, and enumerator-evaluated block faces captured variations in neighborhood context. Residence was coded 1 for living in the inner city area and 0 for residing in the suburban area. The four-item measure (alpha = 0.78) of self-reported neighborhood assessment ranged from 4 (best) to 21 (worst) and captured the quality of the neighborhood as a place to live, positive feelings about living in the neighborhood, attachment to the neighborhood, and neighborhood safety. The five-item measure (alpha = 0.96) of interviewer-assessed housing conditions [37] ranged from 5 (best) to 20 (worst) and tapped the cleanliness, physical condition, furnishings, and exterior of the place of residence as well as an overall assessment at the time of the baseline interview. The five-item (alpha = 0.92) enumerator-evaluated measure of neighborhood conditions [37] also ranged from 5 (best) to 20 (worst) and tapped the conditions of houses, noise levels, air quality, street and road conditions, and yard and sidewalk conditions. These enumerator-evaluations were completed during the process of identifying all eligible housing units in the two strata, and preceded the baseline interviews by several months.

### Healthy Lifestyles

Body mass, physical activity, cigarette smoking, and alcohol dependency captured variations in healthy lifestyles. Body mass (kg/m^2^) was obtained from measured height and weight (with self-reports used for some participants who could not be safely measured), and recoded as 1 for obese (BMI≥30) and 0 for otherwise. Physical activity was measured using the Yale Physical Activity Scale [38] adjusted for seasonal variation (test-retest = .65), which ranged from 0 (low) to 140 (high). Smoking history was measured with two dummy variables, one for being a current cigarette smoker and one for being a former cigarette smoker with each coded 1 for yes and 0 for no (the reference category was never having smoked cigarettes). Alcohol dependency was measured at the one-year follow-up using the four-item CAGE scale (Cut-down, Annoyed, Guilty, and Eye opener) [39], which ranged from 0 (low) to 4 (high). Participants not re-interviewed at the one-year follow-up were coded as 0 (the modal value).

### Attitudes and Beliefs

Religiosity and racial consciousness captured variations in attitudes and beliefs. Religiosity was measured using a five-item scale (alpha = 0.66) adapted from the Fetzer–NIA Working Group [40], which ranged from 5 (high) to 33 (low) reflecting the frequency of private prayer, Bible reading, how religious one felt, spirituality, and the importance of religion. Racial consciousness was measured by asking participants “How often do you think about your race? Would you say never, once a year, once a month, once a week, every day, every hour, or constantly?” [41], with “constantly” coded 1 and all other responses coded 0.

### Morbidity

Self-reports of disease history and whether the participant had been hospitalized in the year prior to baseline captured variations in morbidity. Participants were asked: “Did a doctor ever tell you that you had [X]?”, where “X” included hypertension, diabetes, cancer, chronic obstructive pulmonary disease, congestive heart failure, angina, asthma, arthritis, stroke, or kidney disease. Binary indicators for each disease were coded 1 for positive and 0 for negative responses. Having been hospitalized in the year prior to baseline was coded 1 for yes and 0 for no, capturing recent sentinel health events (morbidity burden) that go beyond the traditional disease history markers [9]).

### Functional Status

Seven measures captured variations in functional status. These included visual and hearing acuity, depressive symptoms, activities of daily living (ADLs), instrumental ADLs (IADLs), mobility limitations, and self-rated health (at baseline). The three-item visual acuity scale (alpha = 0.75) ranged from 3 (excellent) to 15 (poor) and tapped eyesight with glasses, ability to read newsprint, and ability to recognize a friend across the street. Hearing acuity was measured by asking participants to rate their hearing (with hearing aids, if used) as excellent, very good, good, fair, or poor coded as 1–5. Depressive symptoms were measured using the 11-item version of the Center for Epidemiologic Studies Depression scale (alpha = 0.84) [42], which ranges from 0 (no symptoms) to 33 (maximal depressive symptoms). ADLs were measured as the count of difficulties performing seven activities–bathing, dressing, eating, transfers from beds or chairs, walking across a room, getting outside, and using the toilet. IADLs were measured as the count of difficulties performing seven activities–meal preparation, shopping, managing money, using the telephone, heavy housework, managing medications, and getting to places outside of walking distance. Mobility was measured as the count of difficulties performing five activities–walking a quarter mile, going up and down 10 steps, standing for two hours, stooping crouching or kneeling, and lifting and carrying 10 pounds. Self-rated health was measured as described above.

### Analyses

The self-reported health trajectories were identified using a semi-parametric mixture model (*SAS V9.2, Proc Traj*) [31], [32] with standard methods for identifying the optimal number of trajectory groups and the specification of each group distribution. These involved comparing the Bayesian Information Criterion associated with each model, starting with a one-group model and then serially increasing the number of groups, with insignificant linear parameters omitted. This approach identified distinct intra-individual self-rated health trajectories first and then grouped them into more common patterns. The probability that each individual belongs to each group pattern was then estimated using likelihood functions, and each individual was assigned to the group representing their predominant probability. Because self-rated health scores could not be lower than 0 (for death) or higher than 95 (for excellent health), a censored normal distribution was assumed, with the likelihood function including the probability of observing scores of 0 (floor effects) and of observing scores of 95 (ceiling effects). The likelihood function also included a term reflecting when an individual permanently refused to participate any further, with these individuals being censored at that point. All other missing data points were treated as missing at random.

Multivariable multinomial logistic regression analyses were used (*SPSS V20*) with the weighted data to characterize the identified trajectory groups. Given the large number of covariates considered, backwards elimination (with *p≤*0.05 for retention or re-entry) was used to identify the most parsimonious final model and minimize the potential for over-fitting. Model fit was determined using the Cox and Snell pseudo r-squared and the mean correct category prediction probability [43], [44].

## Results

### Descriptive

The mean age at baseline was 56.8 (median = 57; *SD* = 4.4), 42% were men, 28% were divorced or separated, 13% were widowed, and 12% had never been married. The mean educational attainment was 12.5 years (median = 12; *SD* = 2.8), 13% were on Medicaid, 28% had household incomes of $20,000 or less, and 8% were not able to see a doctor when they needed to because of finances. In terms of neighborhood context, 21% lived in the poorer inner city area, the mean score on the self-reported neighborhood quality scale was 10.3 (median = 10; *SD* = 3.4), the mean score on the interviewer-assessed housing conditions scale was 10.3 (median = 10; *SD* = 3.8), and the mean score on the enumerator-assessed block face scale was 11.7 (median = 10; *SD* = 3.0).

Results from the healthy behaviors measures indicated that 41% were obese, the mean score on the Yale Physical Activity Scale was 35.2 (median = 57; *SD* = 4.4), 30% were current smokers and 37% were former smokers, and 19% had CAGE scores of two or more reflecting a positive screen for alcoholism. In terms of attitudes and beliefs, the mean score on the religiosity scale was 13.9 (median = 13; *SD* = 6.1), and 42% thought about their race constantly.

Morbidity results indicated that 63% reported hypertension, 26% reported diabetes, 7% reported having had cancer, 5% reported chronic obstructive pulmonary disease, 5% reported congestive heart failure, 7% reported angina, 10% reported asthma, 45% reported arthritis, 8% reported having had strokes, 5% reported kidney disease, and 18% reported having been hospitalized in the year before their baseline interviews. In terms of functional status, the mean score on the vision scale was 8.3 (median = 9; *SD* = 2.6), the mean score on the hearing question was 2.4 (median = 2; *SD* = 1.1), the mean score on the depressive symptoms scale was 4.9 (median = 4; *SD* = 4.7), the mean ADL score was 0.6 (median = 0; *SD* = 1.4), the mean IADL score was 0.7 (median = 0; *SD* = 1.3), and the mean mobility limitations score was 1.7 (median = 1; *SD* = 2.1). The mean self-rated health score at baseline was 62.7 (median = 80; *SD* = 26.6).

### Identifying the Self-Rated Health Trajectory Groups

The best-fitting model contained four trajectory groups and was quite robust. Of the 998 AAH participants classified (assigned) into the four trajectory groups, their probability for their assigned trajectory group was ≥0.70 for over 95%. In sensitivity analyses (not shown) we re-estimated the trajectory groups without the 51 AAH participants that had <0.70 probabilities for their assigned trajectory group individuals, and the results were unaltered. Therefore, these 51 participants were retained in the analyses that follow. With all AAH cohort participants in the model, the mean correct trajectory group predicted probability was 0.64, reflecting a good fit.

**Figure 1:**
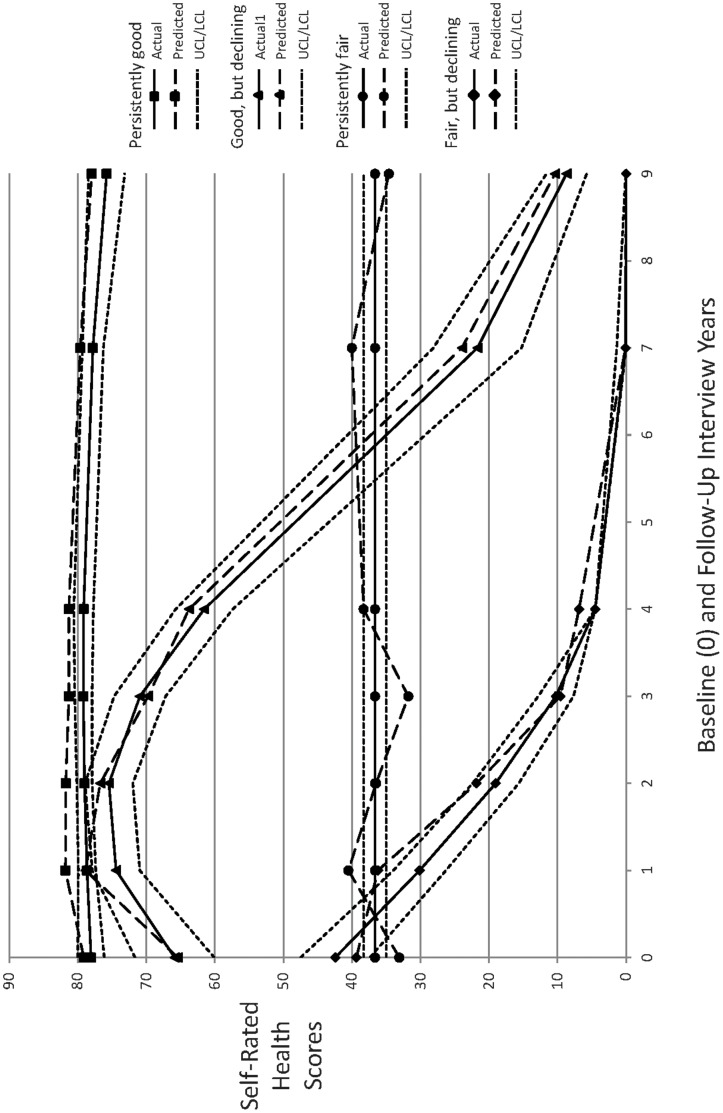
Graphic Portrayal of the Four Identified Self-Rated Health Trajectory Groups. The horizontal axis reflects the baseline (0) and (one-, two-, three-, four, seven-, and nine-year) follow-up interviews and the vertical axis reflects the self-rated health scores. The solid lines for the group trajectories reflect the observed means of the self-rated health scores, the long-dashed lines for the group trajectories reflect the predicted mean self-rated health scores, and the short-dashed lines reflect the upper and lower 95% confidence intervals around those predicted self-rated health trajectories. The persistently good self-rated health trajectory group (N = 537) is indicated with squares, the good but declining self-rated health trajectory group (N = 116) is indicated by triangles, the persistently fair self-rated health trajectory group (N = 267) is indicated by circles, and the fair but declining self-rated health trajectory group (N = 78) is indicated by diamonds.

The four identified self-rated health group trajectories are graphically portrayed in Figure 1. The horizontal axis reflects the baseline (0) and (one-, two-, three-, four, seven-, and nine-year) follow-up interviews and the vertical axis reflects the self-rated health scores. The solid lines for the group trajectories reflect the observed means of the self-rated health scores, the long-dashed lines for the group trajectories reflect the predicted mean self-rated health scores, and the short-dashed lines reflect the upper and lower 95% confidence intervals around those predicted self-rated health trajectories. The persistently good self-rated health trajectory group (N = 537) is indicated with squares, the good but declining self-rated health trajectory group (N = 116) is indicated by triangles, the persistently fair self-rated health trajectory group (N = 267) is indicated by circles, and the fair but declining self-rated health trajectory group (N = 78) is indicated by diamonds. The identification of these four group trajectories is entirely consistent with hypothesis (**H1**), as these were qualitatively distinct from each other and were consistent with the literature, and with our expectations.

The primary explanation of the fair but declining self-rated health group trajectory initially appeared to be mortality. For this trajectory group 21% were dead within one year, 38% were dead within two years, 64% were dead within three years, 70% were dead within four years, and all participants in this group were dead within seven years of baseline. This contrasted with the persistently fair health self-rated health trajectory group, for which no deaths occurred until nine years after baseline, when 7% died.

It is important to note, however, that this apparent mortality-driven trajectory group (fair but declining health) was confounded by the floor effect–those having fair self-rated health at baseline had limited options for declines not involving mortality. That is, while they could have declined from fair to poor health, any subsequent decline would have to have involved death. Indeed, additional analyses (not shown) among those in the fair but declining health trajectory group *who survived to either the third- or fourth-year follow-up interview* revealed average self-rated health scores midway between fair and poor, reflecting a progressively declining self-rated health pattern that preceded their deaths before their next scheduled interview.

Mortality was much less involved in explaining the good but declining self-rated health trajectory group. In this trajectory group there were no deaths in the first three years after baseline, with 6% dying the next year, 35% dying by the seventh year of follow-up, and 47% dying by the ninth year of follow-up. While this contrasted to the persistently good health trajectory group where there were no deaths until the ninth year of follow-up when 1% died, in sensitivity analyses among those *alive at the nine-year follow-up*, the mean self-rated health score among those with good but declining health had dropped to 26 (reflecting fair health). Furthermore, among those *alive at the nine-year follow-up*, the mean self-rated health score for those in persistently good health was 77, while the mean self-rated health score among those with persistently fair health was 38. Taken together, these results clarified that although mortality had played a role in the two declining self-rated health trajectory groups there were substantial declines in the self-rated health scores in these two groups prior to mortality.

### Multivariable Multinomial Logistic Regression Model

Table 1 contains the adjusted odds ratios (*AORs*) obtained from the final multivariable multinomial logistic regression model that differentiated the good but declining, persistently fair, and fair but declining self-rated health trajectory groups from the persistently good self-rated health trajectory group (the reference group). The model fit the data well with a Cox and Snell pseudo r-squared  = 0.59 [44]. Nineteen variables with statistically independent effects were retained in the final multivariable multinomial logistic regression based on the backwards elimination criteria.

**Table 1:** Adjusted odds ratios (AORs) obtained from the multinomial logistic regression of the self-rated health trajectories using persistently good health as the reference group.

Baseline Predictor Variables	Good but Declining Health AORs	Persistently FairHealth AORs	Fair but DecliningHealth AORs	Overallp value
***Demographic Factors***				
Age (in years)	1.101**	1.012	1.146**	.001
Divorced or Separated	0.711	0.727	2.032*	.012
***Socioeconomic Status***				
Education (in years)	0.916	0.938	0.843**	.011
***Healthy Lifestyles***				
Current Smoker	1.563	1.734*	3.065**	.014
Obese	1.313	1.415	0.521	.027
Yale Physical Activity Scale (0 = low, 140 = high)	1.013*	0.994	0.994	.037
CAGE Alcoholism Score (0 = low, 4 = high)	1.241*	1.083	0.776	.013
***Morbidity***				
Angina	4.619***	2.251***	1.595	.009
Arthritis	0.759	2.098**	1.332	.002
Congestive Heart Failure	4.825*	8.406**	26.607***	.001
Diabetes	2.803***	0.889	1.425	.001
Kidney Disease	1.424	1.090	6.951**	.001
Stroke	0.914	1.165	0.235*	.013
Hospitalized in Pre-baseline Year	1.493	1.905*	4.716***	.001
***Functional Status***				
Depressive Symptoms Score (0 = lowest, 33 = highest)	1.078*	1.155***	1.179***	.001
Hearing (1 = excellent, 5 = poor)	0.907	0.898	0.582***	.010
IADL Limitations (0 = low, 7 = high)	0.612*	0.934	1.131	.020
Mobility Limitations (0 = low, 5 = high)	1.340**	1.261**	1.223	.008
Self-Rated Health at Baseline (15 = fair, 95 = excellent)	0.974***	0.941***	0.961***	.001

* = p<0.05; ** = p<0.01; *** = p<0.001.

AAH participants in the good but declining self-rated health trajectory were differentiated at baseline from those in the persistently good health trajectory (the reference group) primarily by their older age, higher alcoholism scores, greater morbidity (angina, congestive heart failure, and diabetes), mobility, and depressive symptom burdens, and their somewhat poorer initial self-rated health. These effects were consistent with the literature, and with our expectations. There were, however, also two marginally statistically independent effects that were not in the expected direction. AAH participants in the good but declining health trajectory were slightly more physically active and had fewer IADL limitations than those in the consistently good health trajectory.

AAH participants in the persistently fair self-rated health trajectory were differentiated at baseline from those in the persistently good health trajectory (the reference group) primarily by their higher smoking rates, greater morbidity (angina, arthritis, and congestive heart failure), mobility, and depressive symptom burdens, having been hospitalized in the year prior to baseline, and their substantially poorer initial self-rated health. These effects were consistent with the literature, and with our expectations.

AAH participants in the fair but declining self-rated health trajectory were differentiated at baseline from those in the persistently good health trajectory (the reference group) primarily by their older age, higher rate of marital dissolution, lower educational attainment, greater morbidity (congestive heart failure and kidney disease), having been hospitalized in the year prior to baseline, and their poorer initial self-rated health. These effects were consistent with the literature, and with our expectations. There were, however, also two statistically independent effects that were not in the expected direction. AAH participants in the fair but declining health trajectory were less likely to have had a stroke, and had better hearing than those in the consistently good health trajectory.

## Discussion

This study was framed by two hypotheses. The first (**H1**) was that several qualitatively distinct intra-individual self-rated health trajectories would be identified in the AAH cohort over the nine years of follow-up. **H1** was fully supported by the data. Four distinct group trajectories–persistently good health, good but declining health, persistently fair health, and fair but declining health–were identified using a semi-parametric group-based mixture modeling strategy [31], [32]. The identification of these four groups was robust, with trajectory group assignment for 95% of the AAH participants involving a predicted probability ≥0.70. Moreover, with all 998 AAH cohort participants in the model, the mean correct trajectory group predicted probability was 0.64, reflecting a good fit.

While the primary explanation of why the declining self-rated health group trajectories (i.e., good but declining, and fair but declining) differed from those that were flat (i.e., persistently good, or persistently fair) initially appeared to be mortality, further analysis revealed that this was not really the case. That is, while both the good but declining and fair but declining health trajectory groups experienced substantially higher rates of mortality than their persistently good or persistently fair health counterparts, there was clear evidence that prior to their death, AAH participants in the declining health trajectory groups had had progressively declining self-rated health.

Our second hypothesis (**H2**) was that the identified self-rated health trajectory groups would be associated with both individual level factors like demographics, socioeconomic status, healthy lifestyles, attitudes and beliefs, morbidity, and functional status, as well as by the context of the two distinct neighborhoods in which the AAH participants resided. **H2** was only partially supported by the data. Multivariable multinomial logistic regression models did successfully differentiate the good but declining, persistently fair, and fair but declining health trajectories from the persistently good health trajectory group (the reference group).

No support, however, was found for the expectation that self-rated health trajectories would partially be explained by the attitudes and beliefs held by the AAH participants, or by the context of the neighborhoods in which they lived. On the one hand, the former may have resulted from the fact that only two measures of participant attitudes and beliefs–religiosity and racial consciousness–were included. While others have shown religiosity to be an important determinant of self-rated health [29], we found no evidence of this using a reliable multi-item scale that has considerable construct validity [40]. While our measure of racial consciousness has been a core item in the *Behavioral Risk Factor Surveillance System* [41] instruments for years, our assumption that it might differentiate self-rated health trajectories among African Americans was also not borne out. Nonetheless, future studies should consider including psycho-social characteristics of study participants, perhaps especially focusing on personality attributes and other factors [28], [29], for further study.

Failure to find support for the role of neighborhood context in self-rated health trajectories [14], [46]–[50], on the other hand, cannot be attributed to insufficient measurement. Our study included geographic catchment area markers as well as multiple item measures of self-reported neighborhood conditions, interviewer-assessed housing conditions, and enumerator-evaluated block faces. None of these measures was found to have significant effects on differentiating one self-rated health trajectory group from another. Thus, in this particular cohort of African Americans from the same major metropolitan area, neighborhood context did not matter for differentiating between self-rated health trajectories.

The main characteristics that differentiated the self-rated health trajectory groups from each other were age, education, smoking, morbidity (angina, arthritis, congestive heart failure, diabetes, and kidney disease), having been hospitalized in the year prior to baseline, depressive symptoms, mobility limitations, and initial self-rated health. As expected, older AAH participants were more likely than their younger counterparts to be in one of the declining self-rated health trajectory groups. Those in the fair but declining health trajectory group had lower levels of educational attainment. Smokers were more likely to start off in fair self-rated health. Participants with congestive heart failure and those who were hospitalized in the year prior to baseline were also more likely to start off in poorer self-rated health, but their self-rated health then declined. Depressive symptoms and mobility limitations also led to AAH participants starting off in poorer self-rated health, or experiencing declines if they started off in good health. Finally, the higher their self-rated health at baseline, the more protected participants were from subsequent health declines.

The seven strengths of this study described in the introductory section allowed it to make two important contributions to the literature, each of which has important implications for future research. First, this study’s within-race analysis of the AAH cohort followed for up to nine years identified four qualitatively distinct self-rated health trajectories. To our knowledge, no previous study has examined self-rated health trajectories separately among African Americans. Thus, our findings call into question the accuracy of prior reports that a single, average self-rated health trajectory for African Americans adequately captures their within-group heterogeneity [14]. Based on these findings, future research using large, nationally representative data sets with sufficient numbers of minorities and multiple data collection points, like the HRS, should be encouraged. Furthermore, such research should consider using discrete group trajectory modeling techniques separately within race and ethnic groups, and then comparing those trajectories across race and ethnic groups. Only then will it become clear whether the self-rated health trajectories observed for African Americans in this cohort are similar to or different from those for Caucasian Americans and Hispanic Americans.

The second important contribution of this study was its use of the Diehr et al. [9] coding strategy for the traditional self-rated health response set. That approach assigns 95 for excellent self-rated health, 90 for very good, 80 for good, 30 for fair, 15 for poor and 0 for decedents. Compared to the traditional 1–5 coding, the Diehr et al. strategy explicitly adjusts for the longstanding recognition that the 1–5 response set is ordinal rather than interval, and that it needs to be transformed (i.e., recoded), as is routinely done in the SF-36 [36]. The Diehr et al. strategy also permits the incorporation of attrition due to death directly into the trajectory estimation process and minimizes the selection bias inherent in most prior reports where those lost to death were censored at their last completed interview. Moreover, this strategy also approximates a simple utility assessment. Given the one- or two-year (or more) gap between the follow-up interview cycles used in most longitudinal studies, rapid declines in self-rated health and functional decline that occur in the six to twelve months before death or after sentinel health events are masked by such censoring [9], [23], [26], [45]. Therefore, future studies should be encouraged to use the Diehr et al. coding strategy and shorter time intervals between interviews.

This study was not without limitations, two of which warrant mention here. The first is that this study was constrained to within-race analyses by the nature of the AAH cohort. Thus, the self-rated trajectories identified here for African Americans cannot be directly compared to what would have been observed for Caucasian Americans or Hispanic Americans. Second, this study relied on static baseline multivariable multinomial logistic regression analyses to differentiate the four identified self-rated health trajectory groups. Future research should consider using time-dependent covariates, because these might facilitate better characterization of the group trajectories.
